# Next-generation sequencing reveals a case of Norrie disease in a child with bilateral ocular malformation

**DOI:** 10.3389/fgene.2022.870232

**Published:** 2022-08-12

**Authors:** Haijun Li, Zhiming Li, Degang Wang, Chuanming Chen, Zhiqiang Chen, Jinhua Wang, Chenxia Xu, Xingsheng Dong

**Affiliations:** ^1^ Prenatal Diagnosis Center, Boai Hospital of Zhongshan, Zhongshan, GD, China; ^2^ Radiological Department, Boai Hospital of Zhongshan, Zhongshan, GD, China; ^3^ Pathology Department, Boai Hospital of Zhongshan, Zhongshan, GD, China; ^4^ Gynaecology Department, Boai Hospital of Zhongshan, Zhongshan, GD, China

**Keywords:** Norrie disease, next-generation sequencing, variant, genetic, NDP

## Abstract

A Norrie disease protein gene (*NDP*) variant, c.174 + 1G > A, was found in a Chinese family through next-generation sequencing and verified with Sanger sequencing. A case of Norrie disease was reported in the first child, and the symptoms were consistent with the results of gene sequencing. The child’s mother, who was pregnant at the time, was found to be a carrier of the identified pathogenic variant. To determine if the fetus carried the same disease-causing variant, prenatal examination and prenatal diagnosis were conducted. The fetus had biocular vitreous abnormalities and complete retinal abnormalities. Genetic testing showed that the fetus had maternally inherited the *NDP* gene variant found in the proband. It was concurrently confirmed that the *NDP* gene variant led to the deletion of 246 bp at the 3′ end of exon 2, resulting in the deletion of the initiation codon and the occurrence of disease. Our study suggests that the diagnosis of rare diseases through next-generation sequencing, combined with prenatal ultrasound and prenatal diagnosis, can help families with known familial genetic diseases. Furthermore, the findings of this study broaden the known genetic spectrum of Norrie disease.

## Introduction

Norrie disease (ND) (OMIM #310600), first reported in 1927 (Norrie, 1927), is an extremely rare X-linked recessive disorder characterized by exudative hyperplasia (Meire et al., 1998) in which blindness can occur in the early postnatal period due to retinal detachment caused by vitreoretinal dysplasia (Andersen and Warburg, 1961; Warburg, 1966). Moreover, 25–50% of patients have extraocular symptoms such as sensorineural deafness and intellectual disability (Fangting et al., 2016; Wu et al., 2017). The pathogenic ND gene is located on chromosome Xp11.3 (ChrX: 4,36,92,969–4,37,17,694) (GRCh37). The *NDP* gene is composed of three exons that encode the 133 amino acids that comprise the Norrin protein (Chen et al., 1993). Norrin is a secreted protein with a cystine-knot motif that activates the Wnt/beta-catenin pathway.

The diagnosis of ND is based on a combination of clinical ocular manifestations and molecular genetic testing. There are no biochemical or functional tests currently used as disease markers. Therefore, identifying novel mutations is essential for clinical and genetic diagnosis.

The purpose of this study was to identify genetic defects in a Chinese ND family and provide clinical guidance for the prevention of birth defects. Next-generation sequencing allowed us to identify a novel *NDP* gene variant (c.174 + 1G > A) in the affected patient in this family, and we established that the variant was inherited from the mother. The pathogenic mechanism of the *NDP* variant c.174 + 1G > A was confirmed to involve the deletion of exon 2, which leads to the deletion of the original start codon and abnormal mRNA expression. In the next pregnancy, the *NDP* gene was sequenced using fetal umbilical vein blood at 27 weeks of gestation for prenatal diagnosis. The fetus in this subsequent pregnancy inherited the same variant, with imaging studies matching the finding. The parents opted to terminate the pregnancy, considering the poor prognosis of their first son.

## Materials and methods

### Patients, editorial policies, and ethical considerations

**FIGURE 1:**
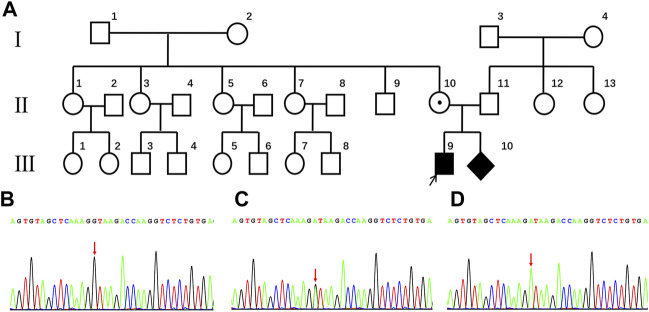
**(A)** Pedigree of the family with Norrie disease. **(B)** partial wild-type *NDP* sequences from the family (Ⅰ-1, Ⅰ-2, Ⅱ-1, Ⅱ-5, Ⅱ-9, Ⅱ-11, Ⅲ-5, and Ⅲ-6). The arrow indicates the proband in this study. **(C)** partial sequences of the family member heterozygous for the c.174 + 1G > A genotype (Ⅱ-10). **(D)** partial sequences of family members hemizygous for the c.174 + 1G > A genotype (Ⅲ-9 and Ⅲ-10).

A three-generation Chinese family was recruited. The family pedigrees are shown in Figure 1A. The woman (Ⅱ-10) had been pregnant for 16 weeks and came to the hospital with her blind son. Because of the blindness of Ⅲ-9, a series of clinical examinations and related genetic testing were carried out. The pregnant women underwent a level Ⅲ ultrasound screening at 25 weeks of gestation. However, no obvious fetal structural abnormalities were found, and fetal eyeball ultrasound monitoring was not performed at that time. Umbilical vein puncture (Daffos et al., 1985) was performed at 27 weeks of gestation. Some other family members of the pregnant women also participated in the study (Figure 1). All participants provided written informed consent. All protocols involving human subjects used in this study were approved by the Ethical Review Committee of the Boai Hospital of Zhongshan. In addition, the research complies with the ethical principles of medical research involving human subjects described in the Declaration of Helsinki.

### Library preparation and next-generation sequencing

Library preparation and next-generation sequencing was performed at the Beijing Genomics Institute (BGI), China. Genomic DNA was extracted according to the manufacturer’s instructions from 300 μl of venous blood collected from the proband (III-9) using the MagPure Buffy Coat DNA Midi KF Kit (Magen, China). To generate short DNA fragments (100–500 bp), the extracted genomic DNA was fragmented using segmentase (BGI, China) and screened with magnetic beads to enrich the fragments with sizes ranging from 280 to 320 bp. Then, the ends were filled, an “adenine” base was added to the 3′ end to facilitate ligation of the DNA fragment to the adapter, and a “thymine” base was added at the 5′ end. A library was constructed using the purified DNA fragments amplified *via* a ligation-mediated polymerase chain reaction (PCR). The library was enriched by array hybridization, according to the manufacturer’s instructions (Roche NimbleGen, United States), eluted, and amplified post capture. The magnitude of product enrichment was estimated using an Agilent 2100 Bioanalyzer. All amplified libraries were subsequently sent to BGI for circularization and sequencing on the MGIseq-2000 platform using a paired-end 100 bp sequencing strategy. The sequencing reads were automatically demultiplexed using the index.

### Bioinformatic analysis

“Clean reads” were generated using the previously published filtering criteria (Wei et al., 2011). These reads were mapped to the human reference genome (hg19) using the Burrows-Wheeler Aligner software (Li & Durbin, 2009). The alignment output files were further subjected to sequencing coverage and depth analyses of the target region, single-nucleotide variants (SNVs), and insertions and deletions (InDels) calling. SNVs and indels were detected using the Genome Analyses Tool Kit (version 3.3), filtered, and assessed using multiple databases, which included the dbSNP (147), 1000 Genome database (phase 3), as well as a database of 100 healthy Chinese adults. The effects of the variants were predicted using scale-invariant feature transform and PolyPhen2. Variants were evaluated according to the American College of Medical Genetics (ACMG) protocol (Richards et al., 2015). Mutations reported in the published studies were screened using the Human Gene Mutation Database (HGMD).

### Sanger sequencing

To validate and detect the novel variant in both the proband (III-9) and the family members (I-1, I-2, II-1, II-2, II-3, and III-2), conventional Sanger sequencing was performed at BGI, China. The primer is shown in Table 1.

**TABLE 1:** The primer for sanger sequencing.


Forward primer	5′-CAG​AAA​GCT​TCA​GCC​CGA​T-3′
Reverse primer	5′-TTG​GAA​AAG​CAC​ACT​ACC​ACT-3′

### Cell lines, culture media, and culture conditions

The HEK293T and HeLa cell lines were purchased from the China Center for Type Culture Collection (China). The cells were cultured with high-glucose Dulbecco’s modified Eagle’s medium (Gibco, United States) containing 10% fetal bovine serum (Gibco, United States) and 1% penicillin–streptomycin (Gibco, United States). Cells were cultured in a constant temperature incubator at 37°C and 5% CO_2_ with saturated humidity.

### Plasmid construction and transfection

To construct a minigene, wild-type and mutant minigenes were inserted into pcMINI and pcMINI-N vectors, respectively. For the pcMINI–*NDP*–wt/mut construct, part of intro 1 (316 bp), exon 2 (381 bp), and part of intro 2 (441 bp) were inserted into the pcMINI vector. The latter contains the universal ExonA-intronA-MCS-introB-ExonB sequence. Cells were then transfected to observe whether the ExonA-Exon2-ExonB shear mode was abnormal.

The minigene construction strategy for pcMINI–N–*NDP*–wt/mut included inserting Exon 2 (381 bp) and part of intro 2 (617 bp) into the pcMINI-N vector. This vector contains the universal MCS-introB-ExonB sequence. After transfection, cells were observed to determine if there was abnormal cutting due to disruptions in Exon B. According to the manufacturer’s instructions (Yeasen Biotech, China), wild-type and mutant vectors were transfected into HEK293T cells using Lipofectamine 2000.

### Minigene splicing assay

Total RNA was extracted from HEK293T and HeLa cells at 48 h post-transfection using TRIzol reagent (Takara, Japan). The extracted RNA was treated with DNase I (Thermo Scientific, United States) and reverse transcribed using the ImProm-II™ Reverse Transcription System according to the manufacturer’s instructions (Promega, United States). To detect alterations in splicing, minigene-specific cDNA was amplified using plasmid-specific primers (Table 2). The PCR products were separated by 12% agarose gel electrophoresis. To characterize the splicing patterns, the PCR products were subjected to Sanger sequencing.

**TABLE 2:** The amplified plasmid-specific primers.

Primer name	Primer sequence
13702-NDP-F	ctg​gag​aga​tct​ctg​gac​cc
13953-NDP-F	agt​ttg​tca​tca​gtg​ctg​gg
15833-NDP-R	atg​gag​agt​gga​ggg​att​gg
16196-NDP-R	agt​caa​cct​tac​atg​ggc​cc
pcMINI-NDP-KpnI-F	ggt​aGG​TAC​Ccg​ttg​ttg​cca​gaa​caa​cat
pcMINI-NDP-EcoRI-R	TGC​AGA​ATT​Cag​aaa​ggc​tca​gac​cac​aaa
pcMINI-N-NDP-KpnI-F	GCT​TGG​TAC​CCT​GTG​CAG​CAG​ATA​CTG​TGA
pcMINI-N-NDP-EcoRI-R	TGC​AGA​ATT​Ctg​gtg​gcc​ttt​taa​gca​tga

### Alternative splicing analysis

RNA (500 ng) was extracted using TRIzol and then reverse transcribed to cDNA using the Illumina TruSeq™ RNA sample preparation kit. RNA-Seq libraries were prepared using the Illumina TruSeq™ RNA sample preparation kit (Illumina, San Diego, CA) and sequenced using the paired-end (150 base paired-end reads) method on an Illumina NovaSeq 6000 platform. Raw data were then quality filtered to generate “clean reads” for further analysis. Clean reads were aligned to the human genome reference (hg19) using the STAR software (Dobin. et al., 2013), with a reference-based assembly of transcripts being performed using HISAT2. Picard was used to compare the results to remove redundancy, with the resultant outputs being screened using Sentieon software to detect SNVs and indels. All previously identified SNVs and indels were determined by using the dbSNP (147) database. The gene expression values were expressed as reads per kilobase of exon per million fragments mapped using Kallisto software. To identify true differentially expressed genes (DEGs), the false discovery rate was used for the rectification of the *p*-values. The significant DEGs (*p* ≤ 0.05, |Log2FC| ≥ 1) were subjected to GO enrichment and KEGG pathway analyses. Protein-to-protein interaction network analyses of DEGs were performed using the STRING database, while the protein–protein interaction network relationship was visualized using Cytoscape software.

## Results

### Clinical characteristics

Having a gravidity of 5 and parity of 1, a 29-year-old woman was referred to the Boai Hospital of Zhongshan at 16 weeks of gestation because her first son suffered from congenital bilateral blindness, without cognitive impairment and deafness. Ultrasonic examination of the affected boy’s eyes showed that the corneas had degenerated, the internal structure of the eyeballs was disordered, and the eyeballs were atrophic. Computed tomography (CT) and magnetic resonance imaging (MRI) scans showed that the brain was normal. However, while the underlying causes of these symptoms and signs have not been identified, in the mother’s family, only the son has this eye disease (Figure 1A).

### Novel likely pathogenic variant identified using whole exome sequencing

The genetic cause of eye disease in the family was determined by performing whole-exome sequencing (WES) on the sample obtained from the proband (III-9). WES generated and aligned 21.47 billion sequenced bases to those found sequences included in hg19. With an average depth of 147.15× and a coverage rate of 99.76%, the bases were mapped to the targeted regions. A total of 23,067 SNVs and indels were detected in 5,700 candidate genes. A hemizygous variant, c.174 + 1G > A, was detected at the *NDP* splicing site. This finding was verified by Sanger sequencing (Figure 1D).

### Sanger sequencing verified the carrier status among family members

Further validation using Sanger sequencing confirmed the heterozygous presence of the variant in the proband’s mother (Figure 1C). In addition, no *NDP* variants were detected in the proband’s maternal grandparents, siblings, or father (Ⅰ-1, Ⅰ-2, Ⅱ-1, Ⅱ-5, Ⅱ-9, Ⅱ-10, Ⅱ-11, Ⅲ-5, and Ⅲ-6; Figure 1B).

### Identified *NDP* variant appears in the fetus

**FIGURE 2:**
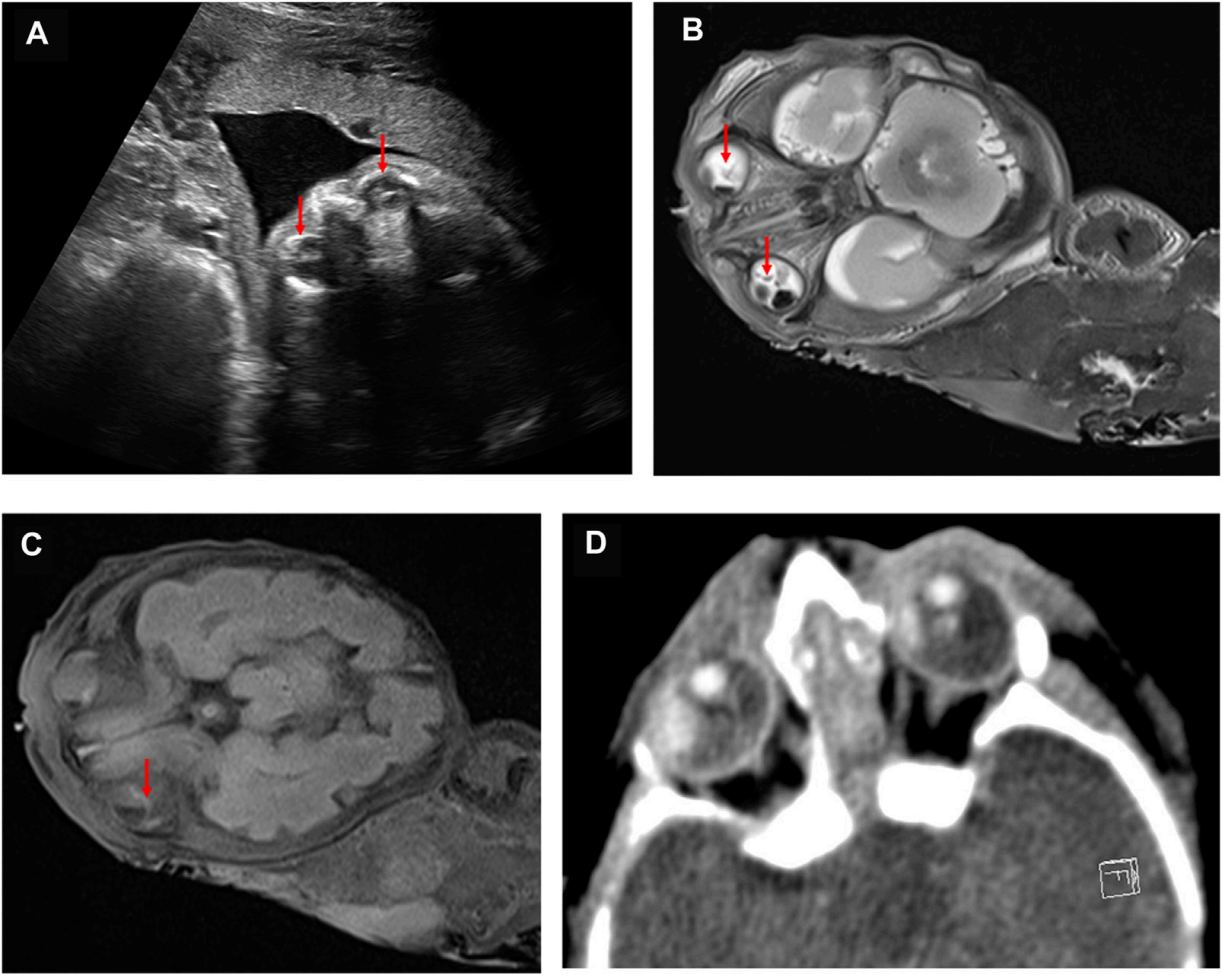
Abnormal fetal imaging results. **(A)** ultrasound showed a hyperechoic mass in both eyes of the fetus at 25 weeks. **(B)** MRI showed abnormally enhanced signals in the bilateral vitreous body of the fetus at 27 weeks. **(C)** as indicated by the arrow, MRI suggests that the fetus at 27 weeks may have a bilateral retinal detachment. **(D)** CT scan showed abnormal bilateral enhancement of the eyeballs in the stillborn infant.

Because ND is an X-linked recessive disease and the expectant mother was a carrier of the *NDP* gene variant, prenatal diagnosis of the fetus was considered. Before conducting the cordocentesis, a level III ultrasound examination was reperformed and a significant exudate was found in the fetal eyeball (Figure 2A). This was accompanied by an MRI examination (Figure 2B); in addition, retinal detachment was found in the fetus by MRI (Figure 2C) and CT (Figure 2D). After cordocentesis, maternal blood contamination was excluded from the cord blood sample. Chromosome karyotype analysis, chromosome microarray analysis (CMA), and *NDP* gene sequencing were subsequently conducted. The chromosome results were 46, XY, and the CMA was normal. The sequencing result indicated the presence of the c.174 + 1G > A variant in the NDP gene.

### Pathology was consistent with Norrie disease

**FIGURE 3:**
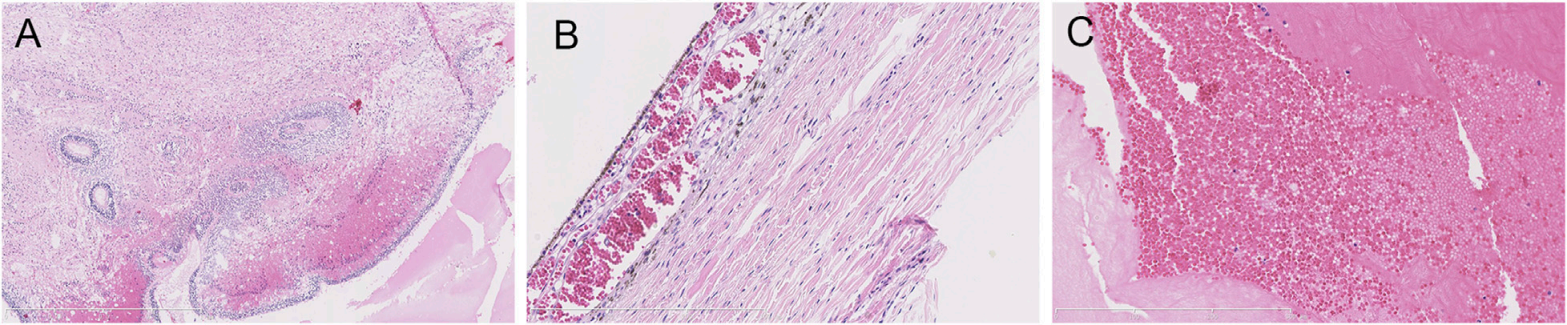
Histopathological examination of the eyeball by HE staining. **(A)** uveal and retinal degeneration and complete retinal detachment (lens, ×4). **(B)** massive bleeding and exudation can be seen under the retina (lens, ×120). **(C)** intraocular fibrous tissue hyperplasia and degeneration and crystal fiber degeneration (lens, ×120).

The parents opted for termination of the pregnancy, considering the poor prognosis of their first son. At 30 + weeks, the stillborn male infant was delivered. With the consent of the hospital medical ethics committee and the parents, an autopsy was performed on the fetus. The autopsy showed that the external appearance of both eyes of the fetus was normal and that the eyeballs were bilaterally symmetrical. However, the corneas of both eyes were transparent, with reduced transparency of both lenses also being noted. Grayish white opacity was observed behind the lenses of both eyes. The histopathological examination of the eyeball showed the degeneration of the uvea and retina, complete detachment of the retina, massive bleeding and exudation under the retina, proliferation and degeneration of intraocular fibrous tissue, and degeneration of crystalline fibers (Figure 3). These features are characteristic of ND. No pathological changes were found in other fetal organs during the autopsy.

### RNA-seq suggested that *NDP* exon 2 was deleted

**FIGURE 4:**
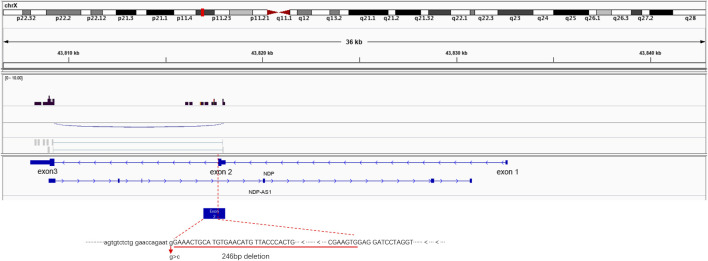
Illustrative examples of variant splicing analysis. RNA-seq was performed to a ×2 sequencing depth to detect the *NDP*-associated RNA. The results were compared to the wild-type transcript sequences. It was found that there was a 246 bp deletion at the 3′ end of *NDP* exon 2.

To observe the *NDP* gene mRNA splicing in the presence of the c.174 + 1G > A variant, RNA-seq was used to detect the splicing of the *NDP* gene. When the c.174 + 1G > A variant was present, exon 1 of *NDP* was not expressed, and the expression of exon 2 was prematurely terminated. The expression of exon 3 was not affected (Figure 4).

### *NDP* c.174 + 1G > A variant leads to a 246-bp deletion in exon 2

**FIGURE 5:**
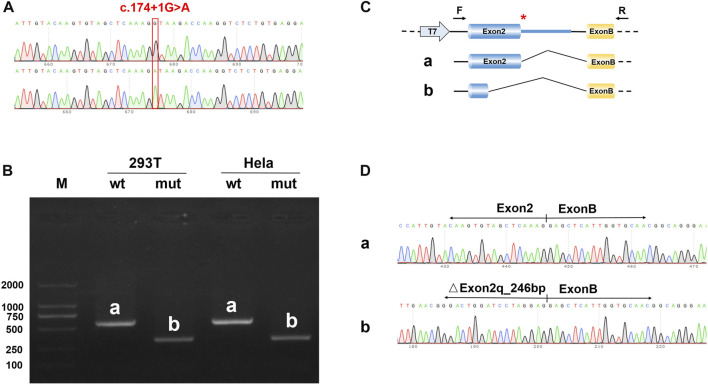
Results of the minigene splicing assay for *NDP* c.174 + 1G > A in HeLa and 293 T cells. **(A)** sequencing map of the minigene constructs, with the WT sequence at the top and the mutant sequence at the bottom. **(B)** electrophoretic map of RT-PCR transcription analysis. The pcMINI–N–*NDP*–wt/mut constructs were transfected into HeLa and 293 T cells, respectively. The RT-PCR results showed that there was a band (band a) associated with wild-type sequences in HeLa and 293 T cells, which was consistent with the expected size (581 bp). The variant resulted in a band (band b) that was smaller than that of the wild-type. **(C)** in the minigene splicing assay, exon 2 (381 bp) and a partial intro 2 sequence (617 bp) were inserted into the pcMINI-N vector. This vector contained the universal MCS-introB-ExonB. The c.174 + 1G > A *NDP* variant was at the 3′ end of exon 2. The wild-type band (band a) shows normal shearing, and the shear mode is Exon 2 (381 bp)-Exon B (57 bp). The variant band (band b) is missing 246 bp at the 3′ end of Exon 2, and the shear mode is Exon 2 (135 bp)-Exon B (57 bp). **(D)** the sequencing results of bands a and b showed that there was a 246 bp deletion at the 3′ end of exon 2.

To detect the splicing effect of the *NDP* c.174 + 1G > A variant on mRNA *in vitro*, two plasmids were constructed. In the first construct, part of intro 1 (316 bp), exon 2 (381 bp), and part of intro 2 (441 bp) were inserted into the pcMINI vector. In the second construct, exon 2 (381 bp) and part of intro 2 (617 bp) were inserted into the pcMINI-N vector. The wild-type and mutant forms of the two plasmids were respectively transfected into HeLa and 293 T cells to observe whether the splicing was abnormal. As shown in Figure 5 and the Supplementary Image, the RT-PCR results showed that two different bands appeared in wild-type and mutant plasmids. After sequencing the two PCR products, it was found that the mutant PCR products had a 246-bp deletion at the 3′ end of exon 2. Given the consistency in results across the two sets of vectors, it was concluded that the c.174 + 1G > A variant disrupts the normal mRNA splicing profile. Following the mutation, a new donor site is generated in the middle of exon 2, which results in a 246-bp deletion at the 3′ end of exon 2. This leads to the deletion of the initiation codon.

## Discussion

ND is caused by variations in the *NDP* gene (Berger et al., 1992). More than 140 point mutations (Zhang et al., 2013), chromosomal rearrangements, frame-shift variants (Wang et al., 2022), nucleotide insertions (Andarva et al., 2018), deletions in the coding region (Zhou et al., 2021) splice site variations, and 5′UTR mutations (Jia and Ma, 2021) have been identified in the *NDP* gene. However, *NDP* gene variants are associated with many retinopathy-related diseases (Dickinson et al., 2006). These diseases, including X-linked familial exudative vitreoretinopathy (FEVR; OMIM #305390), Coat’s disease (OMIM #300216), and retinopathy of prematurity, have similar ocular features to ND. In this study, the proband gradually lost his sight 2 months after birth, while the fetus showed bilateral eyeball deformity by ultrasound examination in the third trimester of pregnancy. Indicating the pathological changes associated with ND, the histopathological examination of the eyes showed bilateral retinal detachment and a lack of retinal nerve cells. Furthermore, the typical family history of congenital blindness in the affected fetus’ biological brother provided an important basis for the diagnosis of the hereditary ophthalmic disease. In the differential diagnosis based on the abnormal ultrasonic images and MRI results, X-linked familial exudative vitreoretinopathy (XL-FEVR) and persistent fetal vasculature (PFV) were the other potential conditions to be considered alongside ND. However, the pathogenic gene *ATOH7* associated with the autosomally inherited condition known as PFV is located on chromosome 10q21.3. Therefore, this disease was not considered further. The pathogenic gene associated with XL-FEVR is located on chromosome Xp11.3; however, disease progression in XL-FEVR is slower than that in ND, with blindness usually manifesting in adolescence (Sızmaz et al., 2015). XL-FEVR is a rare retinal vascular development disorder, which mainly affects retinal angiogenesis. This leads to incomplete vascularization of the peripheral retina and poor vascular differentiation (Gilmour, 2015). While it is often difficult to distinguish ND from PFV and FEVR, genetic analysis may help clinicians accurately diagnose the disease. In this family, the diagnosis of ND was ultimately confirmed by DNA sequencing and ocular histopathological examination.

ND is a complex entity involving severe congenital blindness and progressive deafness in males. According to statistics, more than 60% of ND patients have point variations, while about 20% of patients have *NDP* gene deletions (Huang et al., 2017). However, most *NDP* variations include missense (Meindl et al., 1995), deletion (Schuback et al., 1995), and nonsense variants. Because of the base changes, changes in amino acid structures are often found. This leads to the occurrence of diseases. In this study, a splice site variation was identified in the *NDP* gene. This changed the splicing mode of the mRNA precursor to produce a mature mRNA void of the exon 2 sequence (Figures 4 and 5). This variant, which was inherited from the mother, was novel in the family. All boys who inherited the variant presented pathogenic states.

The *NDP* variant found in this study is a classic splice site variation. According to the ACMG classification standard, the ectopic site of the variant was evaluated as being likely pathogenic. The variant was not found in the HGMD database, suggesting that it is a novel variant. Databases such as dbSNP, OMIM, ESP, Clinvar, 1000 Genomes, and others showed that the pathogenicity of this locus was very high and that the c.174 + 1G > A variant was consistent with being the cause of the common separation disease phenotype in this family. In this study, RNA-seq and minigene results confirmed that c.174 + 1G > A could lead to *NDP* splicing errors resulting in the occurrence of the disease. Splicing variations often occur at the donor (5′) and receptor (3′) ends. These will lead to “exon skipping” and result in mRNA translation errors (Patel and Steitz. 2003). However, in our study, it was found that c.174 + 1G > A affected the normal mRNA splicing structure and resulted in a 246 bp deletion at the 3′ end of *NDP* exon 2. This deletion leads to the absence of the initiation codon. However, the 246 bp deletion did not change the reading frame of exon 3, so the transcription of exon 3 remained unaffected (Figure 4). However, we speculated that c.174 + 1g > A would lead to the loss of the donor site at the 5′ end of intron 2 of *NDP*, and a new donor site would be generated in the middle of exon 2, resulting in the deletion of 246 bp at the 3′ end of exon 2. This deletion would lead to the deletion of the initiation codon of *NDP*, and the translation may start from the downstream AUG, or the protein translation may not initiate. In *NDP*, the next downstream AUG is located at codon 59. Translation may start from this codon, but the translated protein would lack 58 amino acids at the N′-end compared to the wild-type Norrie protein. However, it is unclear how the deletion of exon 2 affects the expression of the Norrie protein and the resultant occurrence of the disease. Maybe it affects changes in the Norrie protein domain or leads to the degradation of the Norrie protein.

In 1996, Fuchs et al. (1996) reported the same variant site as that observed in this study, but identified a G > C transversion variant instead of a G > A transition variant. Their patient presented with bilateral leukocoria and iris dysplasia, as well as posterior synechia and a small cornea in the left eye. The patient showed no signs of deafness or intellectual disability. In this study, the main symptoms of the patients included ocular lesions, such as bilateral leukocoria and atrophy of both eyeballs, but no hearing loss and intellectual disability.

In this study, the function of the c.174 + 1G > A variant was verified through both alternative splicing analysis and a minigene splicing assay. The results suggested that c.174 + 1G > A would lead to a truncated *NDP* exon 2. With a truncated size of 246 bp, this resulted in the expression of a truncated *NDP* gene and the occurrence of ND. However, in this study, it was not verified whether c.174 + 1G > C could also lead to the truncation variation of *NDP* exon 2 or whether the variation size was the same. Therefore, it can be speculated that *NDP* variations at site c.174 + 1 may lead to more serious eye symptoms, but not serious cognitive development and hearing impairment.

As seen in this case, the onset of ND is likely to be a gradual process; we observed abnormalities in the eyeballs of the 27-week fetus, while the brother gradually became blind after birth as described by Wu et al. (2017). However, the occurrence of ND may also be observed in earlier gestational age fetuses. ND is suspected to be responsible for the blindness seen in the child in this family. As a result, in the next pregnancy of the proband’s mother, the doctor should pay attention to the changes in the fetal eyeball by ultrasound in the second trimester of pregnancy. This will assist in finding abnormal fetuses as soon as possible to make a prenatal diagnosis.

## Conclusion

We found a novel ND-associated variant, c.174 + 1G > A, in the *NDP* gene that results in a splicing variation. A new donor site will be generated by this variant in the middle of exon 2, resulting in a 246-bp deletion at the 3′ end of exon 2. This leads to the deletion of the initiation codon. When the diagnosis cannot be made based on clinical characteristics, WES may be a good way to find the root cause of the disease.

## Data Availability

The datasets for this article are not publicly available due to concerns regarding participant/patient anonymity. Requests to access the datasets should be directed to the corresponding author.

## Supplementary material

Supplementary Image S1 Results of the minigene splicing assay for NDP c.174 + 1 G > A in HeLa and 293 T cells. **(A)** sequencing map of the minigene constructs, with WT at the top and mut constructs at the bottom. **(B)** electrophoretic map of RT-PCR transcription analysis. The pcMINI–NDP–wt/mut constructs were transfected into HeLa and 293 T cells, respectively. The RT-PCR results showed that there was a band (band a) associated with wild-type sequences with the expected size (770 bp) in HeLa and 293 T cells. The variant had a band smaller than that of the wild-type, named band b. **(C)** in the minigene splicing assay, partial intro 1 (316 bp), exon 2 (381 bp), and partial intro 2 (441 bp) sequences were inserted into the pcMINI vector which contained universal Exon A-intro A-MCS-intro B-Exon B. The NDP c.174 + 1G > A variant was at the 3′ end of exon 2. The wild-type band (band a) indicates normal shearing, and the shear mode is Exon A (192 bp)-Exon 2 (381 bp)-Exon B (57 bp); the variant band (band b) is missing 246 bp at the3′ end of Exon 2, and the shear mode is Exon A (192 bp)-△ Exon 2 (135 bp)-Exon B (57 bp). **(D)** the sequencing results of bands a and b showed that there was a 246 bp deletion on the right side of Exon 2.
