# Supplementary figures and images for: Next-generation sequencing reveals a case of Norrie disease in a child with bilateral ocular malformation
Source: Front Genet. 2022 Aug 12;13:870232. doi: 10.3389/fgene.2022.870232 (PMC9412000; doi:10.3389/fgene.2022.870232)

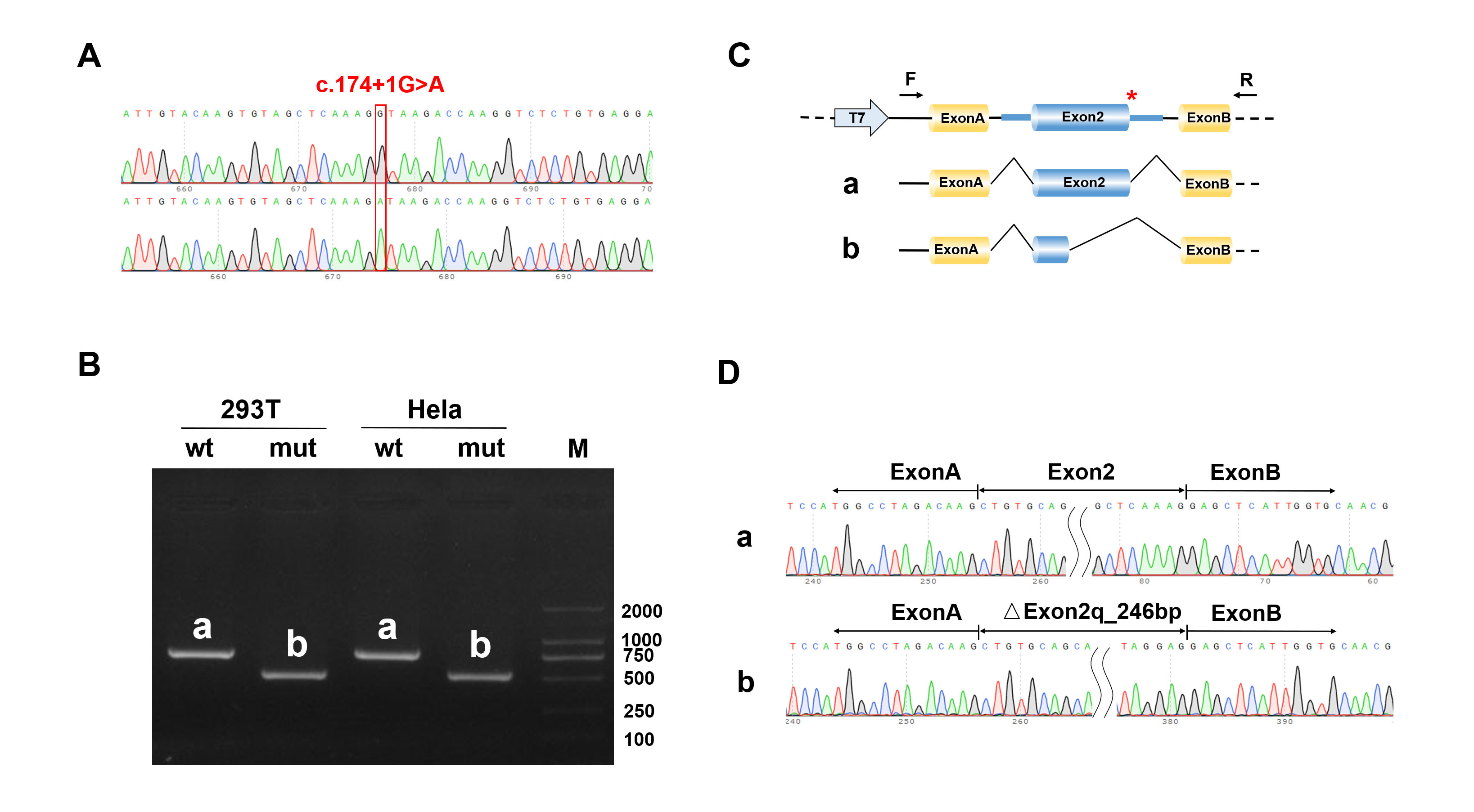

Supplement: Supplementary file 1 [file Image1.JPEG]
